# Transient Osteoporosis of the Hip in the Third Trimester: A Report of a Rare Case

**DOI:** 10.7759/cureus.79267

**Published:** 2025-02-19

**Authors:** Shazia Tariq

**Affiliations:** 1 Obstetrics and Gynecology, Kanad Hospital, Abu Dhabi, ARE

**Keywords:** bone marrow edema, hip pain, multidisciplinary management, pregnancy, transient osteoporosis

## Abstract

Transient osteoporosis of the hip (TOH) is an uncommon, self-limiting condition characterized by acute hip pain and bone marrow edema, which presents in the third trimester of pregnancy. Despite its rarity, TOH can significantly affect maternal mobility and quality of life, highlighting the need for timely recognition and appropriate management. A 28-year-old primigravida presented at 32 weeks of gestation with sudden onset bilateral hip and lower back pain, unresponsive to conservative measures. Laboratory tests ruled out infection and inflammatory causes, and a magnetic resonance imaging (MRI) scan of the hips revealed characteristic bone marrow edema, confirming TOH. Multidisciplinary management included thromboprophylaxis, pain control, and limited weight-bearing. Due to severe pain and compromised mobility, an elective cesarean section was performed at 36 weeks. Postoperatively, her symptoms improved significantly within days. A postpartum dual-energy X-ray absorptiometry scan showed osteopenia, consistent with transient demineralization. At six weeks postpartum, she demonstrated near-complete resolution of symptoms. This case underscores the importance of maintaining a high index of suspicion for TOH in pregnant patients with sudden onset severe hip pain. Magnetic resonance imaging remains the gold standard for accurate diagnosis, and a multidisciplinary approach facilitates safe delivery and effective postpartum recovery. Although the prognosis is favorable, careful follow-up is essential to monitor bone health and mobility in the postpartum period.

## Introduction

Transient osteoporosis of the hip (TOH) is an uncommon, self-limiting musculoskeletal condition characterized by bone marrow edema and the sudden onset of severe pain, typically affecting the hip or pelvis [1]. Transient osteoporosis of the hip is generally more prevalent in men than in women, with a male-to-female ratio of approximately 3:1. In women, the condition is most commonly observed during the third trimester of pregnancy, likely due to altered calcium metabolism and increased weight-bearing stress [2]. The precise pathophysiology remains unclear, but several mechanisms have been proposed, such as transient vascular compromise, hormonal fluctuations, and increased fetal calcium demands [3].

In pregnant women, TOH is often misdiagnosed as common back pain or sciatica, leading to delayed or inadequate management. Early recognition is vital, as severe or persistent symptoms can impair mobility, reduce quality of life, and potentially contribute to complications like pathological fractures [1]. While imaging modalities such as magnetic resonance imaging (MRI) are key to identifying the characteristic bone marrow edema, the rarity of the condition often results in a diagnostic challenge [4].

Management primarily involves conservative measures, including pain control, activity modification, and, in some cases, assistive devices to limit weight bearing on the affected joint [1]. Controversy remains regarding more aggressive pharmacological interventions (e.g., bisphosphonates) in pregnant and breastfeeding individuals because of concerns for fetal and neonatal safety [3].

This case report aims to highlight an instance of TOH manifesting in the third trimester of pregnancy, elucidate diagnostic challenges, and discuss the multidisciplinary approach that facilitated a favorable postpartum recovery. In addition, it addresses potential complications of TOH, such as an increased risk of pathological fractures, joint collapse, and long-term functional impairment, thereby underscoring the importance of early detection and comprehensive management.

## Case presentation

A 28-year-old primigravida with no significant past medical history presented at 32 weeks of gestation with a sudden onset of bilateral hip and lower back pain. Her pregnancy had been uneventful until this point, with normal antenatal visits and standard supplementation, including vitamin D. She reported no trauma, sensory deficits, or systemic symptoms such as fever. Initial clinical evaluation ruled out infection and neurologic compromise; standard laboratory tests (including inflammatory markers and infectious workup) were unremarkable, and serum calcium and vitamin D levels were within normal limits. Despite conservative measures, such as acetaminophen, physiotherapy, and rest, her pain progressively worsened to the extent that she required a wheelchair for mobility.

**Figure 1:**
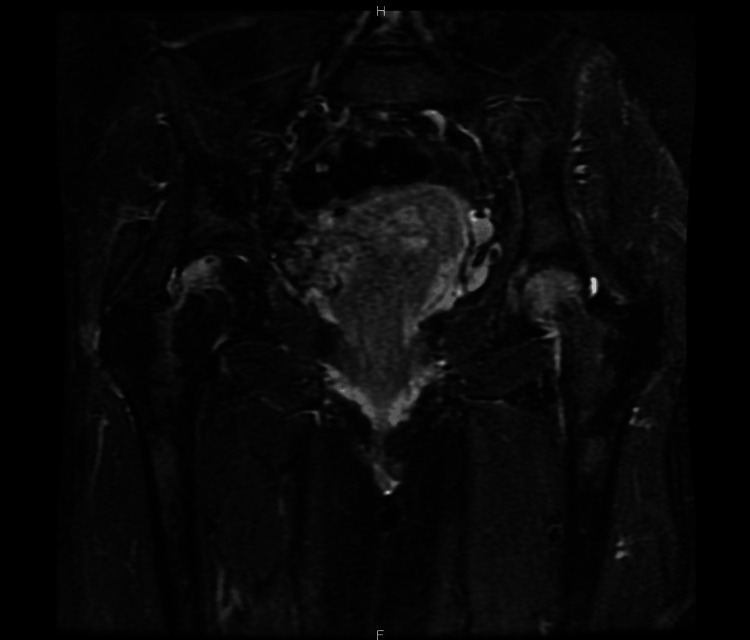
Coronal T2‐weighted MRI of the pelvis Both femoral heads show hyperintense bone marrow edema (arrows), a hallmark of transient osteoporosis of the hip. The patient was at 35 weeks of gestation when the scan was performed.

By 35 weeks of gestation, the patient’s pain was debilitating, limiting her ability to bear weight and perform basic activities of daily living. An obstetric ultrasound showed a normally grown fetus with reassuring biophysical parameters, but her hip pain remained resistant to standard analgesics, including opioids. As there were no obstetric contraindications to further investigation, an MRI of the pelvis and hips was performed. The MRI revealed prominent bone marrow edema in both femoral heads, consistent with TOH. No evidence of lumbar disc pathology or avascular necrosis was identified. An MRI of the pelvis and hips demonstrated prominent bone marrow edema in both femoral heads, consistent with TOH (Figure 1).

A multidisciplinary team, comprising obstetrics, orthopedics, internal medicine, anesthesia, and neonatology, was convened to optimize her management. Due to severe pain and limited mobility, thromboprophylaxis was initiated to mitigate the heightened risk of venous thromboembolism. Urinary catheterization was necessary to reduce the burden of ambulation. After a discussion with the patient about the risks and benefits, an elective cesarean section was planned at 36 weeks. Two doses of antenatal corticosteroids were administered for fetal lung maturity. The surgery was uneventful, and the patient delivered a healthy neonate.

Postoperatively, there was a marked improvement in her pain within three days. She was soon able to ambulate short distances with support, and her need for opioids decreased. At her immediate postpartum visit, a dual-energy X-ray absorptiometry (DEXA) scan was performed, which showed low bone mineral density (T-score in the osteopenic range), consistent with transient osteoporosis and possible pregnancy-related bone demineralization. In view of these findings, she was advised to continue vitamin D and calcium supplementation along with a regimen of gradual weight-bearing exercises and physiotherapy to improve bone strength. Additionally, regarding breastfeeding, the patient was counseled that continuation of breastfeeding was safe and encouraged, as the supplementation and follow-up plan were designed to support both maternal bone health and neonatal well-being.

Following her discharge, contact with the patient was initially sporadic. Recently, however, she reported that she is now walking independently without the need for a walker or crutches, attributing much of her improvement to ongoing physiotherapy sessions. No repeat imaging studies (such as MRI) have been performed because her symptoms have substantially improved and no urgent re-evaluation was deemed necessary. A DEXA scan is also not yet planned; however, the possibility of imaging at around six months postpartum remains under consideration, depending on her clinical course. Her case underscores the importance of continued multidisciplinary follow-up-even when symptoms subside-to monitor for any residual demineralization or functional impairment.

**Figure 2:**
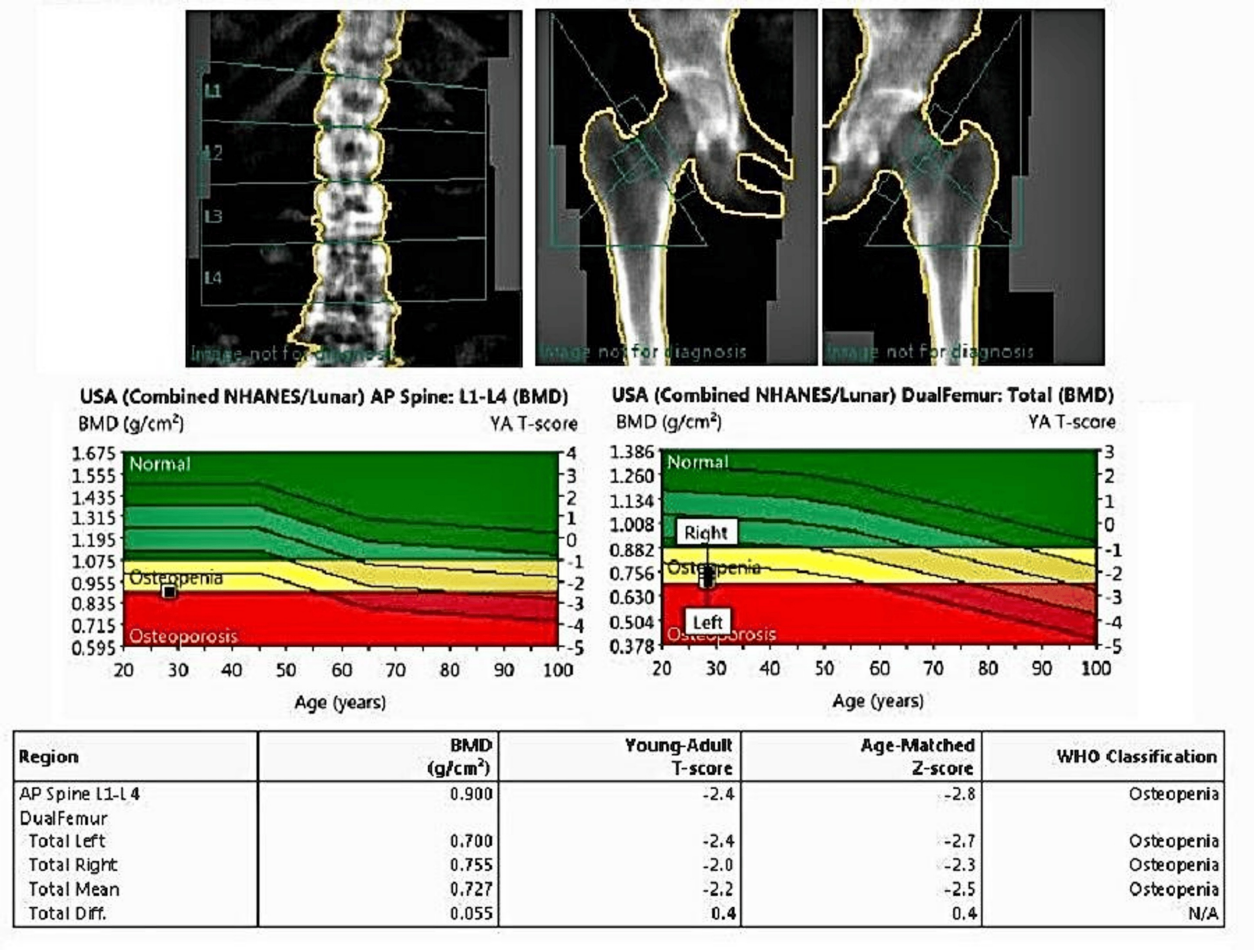
Postpartum DEXA scan demonstrating decreased BMD (yellow‐red zones) consistent with osteopenia. This DEXA report shows decreased bone mineral density in key regions. The AP spine (L1–L4) has a BMD of 0.900 g/cm² with a T-score of -2.4 (Z-score -2.8), indicating osteopenia. The left femur exhibits a BMD of 0.700 g/cm² (T-score -2.4, Z-score -2.7) and the right femur 0.755 g/cm² (T-score -2.0, Z-score -2.3), with an overall mean BMD of 0.727 g/cm² (T-score -2.2, Z-score -2.5). A regional difference of 0.055 g/cm² (T-score difference 0.4) is observed, consistent with transient postpartum demineralization due to increased calcium demands during pregnancy and lactation. DEXA: dual-energy X-ray absorptiometry; BMD: bone mineral density; AP: anteroposterior; NHANES: National Health and Nutrition Examination Survey; WHO: World Health Organization

A postpartum DEXA scan showed low bone mineral density, consistent with transient osteoporosis (Figure 2).

## Discussion

This case highlights the rare yet clinically significant presentation of TOH in a pregnant patient during the third trimester. Although often overlooked, TOH can be distinguished from more common causes of gestational musculoskeletal discomfort by its acute onset of severe pain, progressive functional impairment, and characteristic findings on MRI [5]. Early recognition of the condition is paramount to prevent complications, such as pathologic fractures and prolonged disability [6].

Pathophysiology and risk factors

The precise mechanism underlying TOH remains elusive, but several hypotheses have been proposed. Transient vascular compromise in the femoral head, possibly exacerbated by increased intra-abdominal pressure and diminished venous return during pregnancy, may induce localized bone ischemia [5]. Additionally, fluctuations in sex hormones, particularly estrogen and progesterone, could alter the microcirculation and weaken bone integrity [7]. Rapid fetal growth during the third trimester further increases maternal calcium requirements, potentially contributing to transient demineralization of the maternal skeleton [8]. While vitamin D deficiency has also been discussed as a risk factor, multiple studies have shown inconsistent correlations, suggesting that TOH can occur even in patients with adequate vitamin D levels [9].

Diagnostic considerations

In clinical practice, TOH can be confounded with degenerative joint disease, avascular necrosis (AVN), and neuropathic etiologies such as lumbar disc pathology [5]. Standard laboratory investigations, including inflammatory markers, complete blood count, and metabolic panels, are typically within normal limits, making them useful primarily for ruling out infections or inflammatory arthritides. An MRI is the gold standard diagnostic modality, characteristically revealing bone marrow edema in the affected femoral head without the subchondral collapse seen in AVN [10]. Given the potential severity of pain and the possibility of misdiagnosis, an index of suspicion must remain high whenever pregnant patients present with acute, unexplained hip or pelvic pain.

Multidisciplinary management

Managing TOH in pregnancy requires a multidisciplinary team approach, balancing maternal comfort and safety with fetal well-being. Conservative strategies, such as limited weight-bearing, assistive devices (e.g., crutches or wheelchairs), and appropriate analgesia, are often sufficient for mild to moderate cases [11]. However, when pain is severe, as in this case, more aggressive measures may include hospitalization for pain control and thromboprophylaxis, especially given the increased risk of venous thromboembolism in immobile pregnant patients.

Pharmacological interventions beyond standard analgesics remain a point of debate. Bisphosphonates and calcitonin have been trialed in severe or refractory cases, but concerns persist regarding their safety profiles during pregnancy and lactation [6]. Until more robust data become available, conservative management remains the mainstay of treatment, with multidisciplinary input guiding decisions around the mode and timing of delivery.

Postpartum evaluation and prognosis

A hallmark of TOH is its self-limiting course, with most patients experiencing substantial clinical improvement within weeks to months following delivery [5]. Postpartum bone density assessments are recommended to confirm the resolution of bone marrow edema and to quantify any persistent deficits [8]. In the present case, DEXA demonstrated osteopenia, reinforcing the need for continued monitoring. Prolonged breastfeeding can further influence bone remodeling processes, although evidence suggests that maternal bone density recovers upon cessation of lactation [12]. Accordingly, long-term follow-up, inclusive of repeat imaging and bone density assessments, helps ensure that transient demineralization fully resolves and mobility is restored.

## Conclusions

Transient osteoporosis of the hip is a rare but meaningful differential diagnosis for severe, acute hip pain in the third trimester of pregnancy. This case underscores the importance of prompt MRI evaluation, multidisciplinary care, and individualized management strategies to mitigate functional limitations. Although the prognosis is generally favorable, especially after delivery, heightened awareness and timely intervention are key to preventing complications. Further research is needed to elucidate the optimal therapeutic pathways, particularly regarding pharmacological agents in pregnancy and lactation, to optimize outcomes for both mother and child.
